# Examining Working Memory Performance in Adult Psychosomatic Inpatients

**DOI:** 10.3389/fpsyg.2021.589809

**Published:** 2021-08-16

**Authors:** Judith Held, Laura Ramadani, Andreea Vîslă, Volker Köllner, Peter Hilpert, Christoph Flückiger

**Affiliations:** ^1^Department of Psychological Interventions and Psychotherapy, University of Zurich, Zurich, Switzerland; ^2^Department of Psychosomatic Medicine, Rehabilitation Center Seehof, Federal German Pension Agency, Teltow, Germany; ^3^Research Group Psychosomatic Rehabilitation, Charité – University Medicine Berlin, Berlin, Germany; ^4^School of Psychology, University of Surrey, Surrey, United Kingdom

**Keywords:** working memory, repetitive negative thinking, anxiety, psychosomatic, rehabilitation

## Abstract

**Background:**

There is cumulating evidence that working memory (WM) processing is impaired in individuals suffering from a psychosomatic and a psychological disorder. However, it is unclear how repetitive negative thinking (RNT), depressive symptoms, and patient characteristics (i.e., age and incapability to work) contribute to WM impairments. The present study examines how these factors affect WM performance in highly distressed adult psychosomatic inpatients.

**Methods:**

Seventy-six inpatients (*M*_age_ = 52.7, SD = 8.4) from a psychosomatic rehabilitation clinic performed a two-block WM updating task, with accuracy and reaction time as indicators of WM functioning.

**Results:**

Multivariate mixed effect model results show that accuracy and reaction time significantly decreased from WM Block 1 to WM Block 2. Higher levels of RNT, more severe depressive symptoms and higher age were associated with worse WM accuracy in Block 1. None of these variables were significantly associated with WM reaction time (in Block 1).

**Conclusion:**

From a clinical perspective, the results suggest that screening for the presence of high RNT levels, severe depressive symptoms or higher age may help to identify patients with impaired WM functioning and to intervene on these important patient characteristics early in the rehabilitation process.

## Introduction

Mental disorders are common in individuals suffering from chronical physical conditions, with symptoms of depression being most prevalent (Deschênes et al., 2015). Particularly psychosomatic diagnoses have been found to be the most common cause of disability pensions in the past years (Dannenberg et al., 2010). As a result, early retirement or extensive psychosocial and vocational rehabilitation programs may be necessary in order to restore work capacity and well-being (Köllner, 2014; Linden, 2014).

Connected to this notion, cognitive functions, such as the working memory (WM) have been found to be impaired in a range of psychosomatic and mental disorders. The WM is a prominent system central to cognitive functioning with limited capacity and is, therefore, restricted in how much information can be actively held in WM (Eriksson et al., 2015). Recent evidence indicates that the WM is frequently impaired in psychosomatic disorders (Haley et al., 2008; Schmitz et al., 2008) as well as in mental disorders, including depression (Snyder, 2013) and anxiety (Moran, 2016). For example, Wagner et al. (2006) found cognitive impairments in 24% of the patients from a rehabilitation clinic who suffered from psychosomatic and mental disorders. These results highlight that especially in patients with psychosomatic disorders, impairments in WM functioning may be even more disabling, a fact that might further impact the rehabilitation process (Wagner et al., 2006; Köllner, 2014).

In the past decades, research has identified various factors contributing to WM impairments, one of them being repetitive negative thinking (RNT; Snyder, 2013; Moran, 2016; Zetsche et al., 2018) which has been found in a range of mental disorders (McEvoy et al., 2013) as well as in psychosomatic populations (Trick et al., 2016). RNT is characterized by repetitive thinking about negative topics which is experienced as difficult to control (Ehring and Watkins, 2008). It has been proposed that RNT occupies available WM capacities by acting as a dual task and thereby leading to impaired WM functioning (Beckwé et al., 2014; Zetsche et al., 2018). Indeed, WM impairments have been consistently observed in individuals suffering from a mental disorder when engaging in RNT (Watkins and Brown, 2002; Joormann and Gotlib, 2008; Gustavson and Miyake, 2015; Sari et al., 2016). However, to date, no study has investigated the effect of RNT of WM performance in psychosomatic populations.

In addition, a growing amount of evidence suggests that depressive symptomatology might be another relevant factor contributing to WM impairments (Wagner et al., 2006; Snyder, 2013). The majority of studies investigated outpatients with depressive disorders and found significant associations between depressive symptoms and WM deficits (Christopher and MacDonald, 2005; Joormann and Gotlib, 2008). However, to the best of our knowledge, only one study investigated the effects of depressive symptoms on WM performance in psychosomatic inpatients (Wagner et al., 2006). The results of this study indicated that subjective memory impairments were most pronounced in psychosomatic patients with depressive symptoms.

Taken together, although there is substantial evidence suggesting that WM impairments in individuals with mental disorders may result from interference with cognitive processes (i.e., RNT) and severe clinical symptoms (i.e., depression) there seems to be a lack of studies investigating these concepts in patients with psychosomatic disorders. The majority of studies investigated outpatients (Joormann and Gotlib, 2008) and it still unclear whether WM functioning is affected in psychosomatic inpatients or not. As pointed out above, many psychosomatic patients are adult individuals who are unable to work and therefore, considering these characteristics (age and inability to work), it might further be of importance to investigate WM performance. Moreover, the majority of participants in the past studies were either young adults (30–40 years) or older patients (70 years) and therefore, we have limited information about how these mechanisms manifests in adults more generally (Mattay et al., 2006). Finally, few studies assessed WM performance in more than one WM block (i.e., assessing more than 15 min; Sari et al., 2016) and one advantage of using two blocks over the standard use of one block is the ability to examine the WM performance stability across the two block (Scharfen et al., 2018).

## The Current Study

This study seeks to investigate WM performance in psychosomatic inpatients who referred to a psychosomatic rehabilitation clinic. All participants performed a WM task which consisted of two blocks (WM Block 1 and 2) lasting in total 30–40 min. *Block* denotes a complete WM task which is usually performed by participants as a single task (e.g., Sari et al., 2016). WM performance was measured as accuracy and reaction time. First, we explore the change of accuracy and reaction time in WM from Block 1 to Block 2 (Research Question 1; RQ1). Second, we explore the potential relevance of cognitive factors (RNT), depressive symptom severity, age and incapability to work on accuracy and reaction time (RQ2).

## Materials and Methods

### Participants

Total sample included seventy-six inpatients (50 females) of the psychosomatic Rehabilitation Clinic Seehof in Germany which is a specialist clinic for cardiological, psychosomatic and psycho-cardiological rehabilitation (for more information, see Supplementary Material). During the weekly introduction seminar for new patients, information about the WM assessment was given and interested patients could sign up. Participation was not compensated in any form.

Patients were 52.7 years old (SD = 8.4, range: 30–71 years) and stayed on average 39 days (SD = 8.2) in the clinic. In total, 60.5% indicated being incapable of work at intake. The majority of patients was admitted with an affective disorder (80%), followed by anxiety disorders (41%), adjustment disorder (20%), and somatoform disorder (22%), diagnosed according to the ICD-10 (World Health Organization, 2004). Psychological disorders co-occurred in 75% of the patients. 83% had at least one somatic disorder, the most prevalent somatic disorders included hypertension (22.3%), obesity (14.4%), tinnitus (6.5%), and migraine (5%).

### Ethical Standards

This study protocol was approved by the ethical committee of the Federal Medical Association (“Landesärztekammer”) of Brandenburg, Germany (AS119(bB)/2018). Participants were not compensated for participation.

### Questionnaires

The Perseverative Thinking Questionnaire (PTQ) is a 15-item long content-independent measure of RNT (Ehring et al., 2011). The PTQ mean score in the present study was 38.6 (SD = 11.4, range 4 to 59), with higher scores representing a greater tendency to engage in RNT. Internal consistency in the present study was excellent (Cronbach’s α = 0.95).

The Beck Depression Inventory (BDI) is based on 21 items and measures depression Severity (Keller et al., 2008). The BDI was assed at intake (*M* = 26, SD = 12.1, range: 1–52). Internal consistency in the present study was excellent (BDI_intake_: Cronbach’s α = 0.93).

In order to assess the subjective workload after each WM Block, the National Aeronautics and Space Administration Task Load Index (NASA-TLX; Pfendler, 1991) was administered. Five items assessing mental, physical and temporal demands, effort and frustration were rated on a Likert-scale ranging from 1 (“not at all”) to 5 (“totally agree”). Furthermore, for the purposes of the present study, the NASA-TLX was extended (extended NASA) with additional seven items (alert, interested, attentive, nervous, jittery, afraid, and distressed) of the Positive and Negative Affect Schedule (PANAS; Krohne et al., 1996). The items were rated on a five-point Likert-scale ranging from 1 (“not at all”) to 5 (“very much”).

### Working Memory Task

A two-block numerical updating WM task (Oberauer et al., 2000) was used. In total, each WM block consisted of 150 trials and the two blocks were two alternate versions of the same updating task. The WM task was self-paced (for more information, see Supplementary Material). The updating task included math problems presenting three or four boxes with a number in each box (e.g., 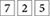).

In a next step, in one of the boxes, an arithmetic operation appeared (e.g., 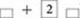) and participants were asked to perform the arithmetic operation and type in the result (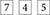 in the present example). After eight additional arithmetic operations participants were asked to type in the last number they recall (see Held et al., 2020 for a more precise description of the task).

### Procedure

Participants performed two WM blocks (Block 1 and 2) and filled out questionnaires at three time points (T1: before WM Block 1, T2: between WM Block 1 and 2, T3: after WM Block 2). First, participants filled out the informed consent form for study participation, as well as the PTQ and the extended NASA (T1). Next, the WM task was explained verbally by the experimenter and participants completed two practice trials. Afterward, the experimenter left the room and WM Block 1 was started. After Block 1, participants were asked to fill out the extended NASA (T2). Next, a text was presented on the screen telling participants to do their best and focus (focus reminder). By clicking a key, participants started WM Block 2. After the completion of WM Block 2, participants were again asked to fill out the extended NASA (T3). Afterward, the experimenter thanked participants for their participation and answered open questions.

### Statistical Analysis

The main goal of the current study was to examine how RNT, depressive symptom severity and patient characteristics (age, duration of incapability of work) influence WM accuracy and reaction time in Block 1 as well as the change from Block 1 to Block 2. Multivariate multilevel models with a random intercept and a fixed slope were used to simultaneously analyse the two outcome variables (accuracy and reaction time) as well as the nested data structure (repeated measures nested in individuals). The intercept for the WM Blocks was centered at Block 1 and the slope indicates the average change in accuracy or reaction time from Block 1 to Block 2. The equations for the multilevel model and more details about the associations between all variables of interest can be found in the Supplementary Material. For all analyses we used *nlme* package (Pinheiro et al., 2021) for multilevel modeling in R (R Core Team, 2021).

## Results

Descriptive statistics and correlations of key variables are displayed in Supplementary Table 1A. To analyse the subjective workload as well as the emotional experiences during the assessment, each item from the extended NASA was analyzed individually in univariate multilevel models with a random intercept. Results indicate that from T1 (before Block 1) to T2 (after Block 1), patients felt significantly more alert [*t* (150) = 2.97, *p* = 0.003], more interested [*t* (150) = 2.35, *p* = 0.01], less afraid [*t* (150) = −2.83, *p* = 0.005], less distressed [*t* (150) = −2.79, *p* = 0.005], but also more jittery [*t* (148) = 2.10, *p* = 0.04; Supplementary Table 2A]. After Block 2 at T3, patients’ rating of physical demands and level of effort were significantly higher in comparison to T1 [physical demands: *t* (75) = 2.19, *p* = 0.03; effort: *t* (75) = 2.55, *p* = 0.01].

### Change in Accuracy and Reaction Time From Block 1 to 2 (RQ1)

Results indicate a significant decrease in accuracy from Block 1 to Block 2 [*t* (225) = −2.05, *p* = 0.04], as well as in reaction time [*t* (225) = −2.12, *p* = 0.03; Table 1]. In other words, patients became less accurate but increased their answering speed from Block 1 to Block 2.

**TABLE 1:** Results of the multivariate growth model with random intercepts and fixed slope.

Fixed effects		Accuracy				Reaction time			
		γ	SE	*t*	*p*	γ	SE	*t*	*p*
Empty model	Intercept	78.3	(1.65)	47.2	<0.001	3,541.8	(126.35)	28.03	<0.001
	Block	−2.2	(1.07)	−2.05	0.040	−131.57	(48.63)	−2.70	0.007
Model PTQ	Intercept	78.3	(1.61)	48.5	<0.001	3,541.7	(125.25)	28.27	<0.001
	Block	−2.21	(1.08)	−2.05	0.04	−131.57	(48.94)	−2.68	0.008
	PTQ	−0.36	(0.14)	1.61	0.01	17.43	(11.10)	1.56	0.11
	PTQ × Block	0.08	(0.09)	0.91	0.35	−1.06	(4.34)	−0.24	0.80
Model BDI	Intercept	78.3	(1.55)	50.3	<0.001	3,541.8	(125.78)	28.15	<0.001
	Block	−2.21	(1.07)	−2.06	0.039	−131.57	(48.94)	−2.68	0.008
	BDI	−0.48	(0.12)	−3.73	<0.001	13.09	(10.45)	1.25	0.21
	BDI × Block	0.11	(0.08)	1.32	0.18	0.88	(4.06)	0.21	0.82
Model Age	Intercept	78.3	(1.63)	47.9	<0.001	3,541.8	(122.89)	28.81	<0.001
	Block	−2.21	(1.03)	−2.13	0.033	−131.57	(47.86)	−2.74	0.006
	Age	− 0.48	(0.19)	−2.49	0.013	28.07	(14.71)	1.90	0.057
	Age × Block	0.33	(0.12)	2.66	0.0082	10.59	(5.72)	1.84	0.065
Model WI	Intercept	83.3	(5.2)	16.0	<0.001	3,323.4	(397.86)	8.35	<0.001
	Block	−3.27	(3.4)	−0.96	0.33	−109.6	(153.4)	−0.71	0.47
	WI	−1.62	(1.59)	−1.01	0.30	70.60	(121.94)	0.57	0.56
	WI × Block	0.34	(1.04)	0.32	0.74	−7.09	47.02	−0.15	0.88

**Random effects**		**Accuracy**			**Reaction time**			**Residual**	
		**Variance**	**SD**		**Variance**	**SD**		**Variance**	**SD**

Empty model	Intercept	162.40	12.74		1 108 679	1,052.93		43.36	6.60
Model PTQ	Intercept	149.83	12.24		1 072 385	1,035.56		43.19	6.57
Model BDI	Intercept	136.90	11.7		1 082 182	1,040.27		42.66	6.53
Model Age	Intercept	157.13	12.53		1 032 902	1,016.31		39.85	6.31
Model WI	Intercept	160.15	12.65		1 103 816	1,050.62		43.61	6.60

*Model names are organized according to research questions (RQ; e.g., Model 1 examines RQ1); γ = predictor; SE = standard error; *t* = test statistic of the multivariate growth model; SD = standard deviation; PTQ = Perseverative Thinking Questionnaire; BDI = Beck Depression inventory; WI = duration of work incapability during the last year before the clinic stay.*

### Associations of PTQ, Depressive Symptoms (BDI), Age and Incapability of Work With Accuracy and Reaction Time (RQ2)

Next, PTQ, BDI, age and incapability of work were separately integrated as Level-2 characteristic into the Models (see Table 1). Results showed a significant main effect of PTQ on accuracy [i.e., PTQ indicated lower accuracy at Block 1, *t* (221) = −2.52, *p* = 0.01], but not on reaction time [*t* (221) = 1.56, *p* = 0.11]. Furthermore, depressive symptoms measured with the BDI were significantly associated with accuracy in Block 1 [i.e., depressive symptoms indicate lower accuracy, *t* (221) = −3.73, *p* > 0.001], but not with reaction time [*t* (221) = 1.25, *p* = 0.21]. There were no significant Block by PTQ or Block by BDI interactions for neither accuracy nor reaction time (i.e., no moderation of PTQ or BDI on change from Block 1 to 2). Higher age was significantly associated with decreased accuracy at Block 1 [*t* (221) = −2.49, *p* = 0.01], but not with reaction time [*t* (221) = 1.90, *p* = 0.06]. However, as indicated by a Block by age interaction [*t* (221) = 2.66, *p* = 0.008], older adults performed comparably good *at Block 2* as younger adults. Last but not least, duration of incapability of work was neither associated with accuracy [*t* (221) = −1.01, *p* = 0.30] nor reaction time [*t* (221) = 0.57, *p* = 0.56] at Block 1. No significant Block by incapability of work interaction were obtained for accuracy and reaction time.

## Discussion

There is cumulative evidence suggesting that WM performance is impaired in individuals suffering from a psychosomatic or mental disorder (Snyder, 2013; Moran, 2016). The present study examines WM performance in a two-block WM task (Block 1 and Block 2) in highly distressed psychosomatic inpatients and results indicate that RNT, depressive symptoms and patient age is associated with lower WM performance.

First, we observed a significant decrease in accuracy as well as in reaction time over the course of the WM task (RQ1), meaning that patients made more errors but at the same time became faster in their answering speed from Block 1 to Block 2. One possible explanation for this finding might be the nature of the task itself: Initially, when confronted with an unknown WM task, patients may have perceived the situation as highly stressful (Eysenck et al., 2007). However, during Block 1, patients may have gotten familiar with the task and gained confidence to increase their answering speed. Indeed, participants indicated feeling less distressed and less afraid in the self-reports. When the WM task continued in Block 2, patients indicated an increase in physical task demands and a higher level of effort. As a result, patients may have made more errors. However, since this is a starting point of understanding WM processing across two blocks, future research is needed to better understand WM functioning across two- or more blocks.

With regard to RQ2, we found that patients who reported higher RNT scores as well as more severe depressive symptoms showed lower accuracy scores in Block 1. This is in line with recent meta-analytic evidence indicating that individuals with high RNT seem to have impaired WM performance, specifically, in difficulty in discarding no longer relevant material from WM (*k* = 94; Zetsche et al., 2018). However, Zetsche et al. (2018) only obtained low correlations between measures of psychopathology (i.e., anxiety and depression) and WM deficits, but hypothesize that they may have underestimated this relation by summarizing correlation coefficients within diagnostic groups, resulting in limited variance. Indeed, our results indicate that WM impairments may even be greater in psychosomatic patients with high depressive symptoms which confirms previous work in this field (Christopher and MacDonald, 2005; Wagner et al., 2006). Taken together, the results highlight that RNT and depressive symptoms seems to be relevant factors affecting WM performance in psychosomatic inpatients.

Furthermore, we found that older patients made significantly more errors (i.e., lower accuracy) in Block 1. These results are in line with prior findings which indicate a progressive effect between age and mental disorders, leading to a decreased WM performance (Snyder, 2013). Therefore, particularly in older employees suffering from psychosomatic disorders, WM impairments might be particularly challenging. Moreover, our results indicate that duration of work incapability prior to clinic admission did not affect WM performance. In a similar study, Wagner et al. (2006) did not find differences between psychosomatic patients with or without memory impairments with regard to capability to work at discharge. Taken together, the present results highlight the need to consider multiple factors, such as age, symptom severity and their potential interaction when assessing WM functioning across adulthood.

There are several limitations to consider when interpreting the results of this study. First, the recruitment context might impact the representativeness of the sample: Participation in the WM assessment was solely voluntary; therefore, it is possible that only those not so severely impaired signed up for participation. By assessing WM functions as a standardized part of intake and discharge assessments in clinics, it could further help shaping the treatment according to the patients’ cognitive abilities (Beblo, 2002). Moreover, integrating cognitive training programs focusing on compensating and improving WM functioning within the psychosomatic rehabilitation context could be beneficial (Wagner et al., 2008). Second, we did not recruit a comparison group (i.e., ambulatory or healthy participants). Hence, in order to be able to disentangle how RNT influences WM functioning, it would be helpful to investigate individuals with more variability in RNT and/or (sub-)clinical symptoms. Third, the majority of the patients were female and a contrast between gender would be underpowered based on the obliquely distributed characteristic and the limited sample size. Therefore, we were not able to rule out potential gender differences for this particular WM task. Forth, even though we used a WM task whose practicality was previously tested in patient samples, fatigue effects (e.g., Li et al., 2020) and further aspects of the self-paced format could have an impact on the particular WM task (e.g., Bailey, 2012).

To conclude, the present study investigated WM performance in an adult psychosomatic inpatient population and found that RNT, depressive symptom severity and age significantly is associated with lower WM functioning. Assessing WM functioning may help to better personalize treatment planning in psychosomatic populations in respect to their challenges in psychosocial functioning and work conditions.

## Data Availability Statement

The raw data supporting the conclusions of this article will be made available by the authors, without undue reservation.

## Ethics Statement

The studies involving human participants were reviewed and approved by the ethical committee of the Federal Medical Association (“Landesärztekammer”) of Brandenburg. The patients/participants provided their written informed consent to participate in this study.

## Author Contributions

All authors were involved in conceptualizing and writing up this study report. JH, LR, VK, and CF were responsible for the study conceptualization. JH performed the data analysis and was the major responsible in writing up the manuscript. LR was responsible for the data collection and preliminary analyses. CF and VK were the principal investigators. CF, AV, PH, and VK provided advice for the data analysis and study report.

## Conflict of Interest

The authors declare that the research was conducted in the absence of any commercial or financial relationships that could be construed as a potential conflict of interest.

## Publisher’s Note

All claims expressed in this article are solely those of the authors and do not necessarily represent those of their affiliated organizations, or those of the publisher, the editors and the reviewers. Any product that may be evaluated in this article, or claim that may be made by its manufacturer, is not guaranteed or endorsed by the publisher.

## References

[B1] Bailey H. (2012). Computer-paced versus experimenter-paced woring memory span task. Are they equally reliable and valid?. *Learn. Individ. Diff.* 22 875–881. 10.1016/j.lindif.2012.06.004

[B2] Beblo T. (2002). Die Relevanz neuropsychologischer Untersuchungen bei Depression im Alter. *Z. Geronto. Geriat.* 35 111–117. 10.1007/s003910200015 12080574

[B3] Beckwé M. Deroost N. Koster E. H. W. De Lissnyder E. De Raedt R. (2014). Worrying and rumination are both associated with reduced cognitive control. *Psychol. Res.* 78 651–660. 10.1007/s00426-013-0517-5 24077776

[B4] Christopher G. MacDonald J. (2005). The impact of clinical depression on working memory. *Cogn. Neuropsychiat.* 10 379–399. 10.1080/13546800444000128 16571468

[B5] Dannenberg A. Hofmann J. Kaldybajewa K. Kruse E. (2010). Rentenzugang 2009: Weiterer Ansteig der Zugänge in Erwerbsminderunsrente wegen psychischer Erkrankungen. *RVaktuell* 57 283–293.

[B6] Deschênes S. S. Burns R. J. Schmitz N. (2015). Associations between depression, chronic physical health conditions, and disability in a community sample: A focus on the persistence of depression. *J. Affect. Disord.* 179 6–13. 10.1016/j.jad.2015.03.020 25841076

[B7] Ehring T. Watkins E. R. (2008). Repetitive negative thinking as a transdiagnostic process. *Int. J. Cogn. Therapy* 1 192–205. 10.1521/ijct.2008.1.3.192

[B8] Ehring T. Zetsche U. Weidacker K. Wahl K. Schönfeld S. Ehlers A. (2011). The Perseverative Thinking Questionnaire (PTQ): Validation of a content-independent measure of repetitive negative thinking. *J. Behav. Therapy Experiment. Psychiatry* 42 225–232. 10.1016/j.jbtep.2010.12.003 21315886 PMC3042595

[B9] Eriksson J. Vogel E. K. Lansner A. Bergström F. Nyberg L. (2015). Neurocognitive Architecture of Working Memory. *Neuron* 88 33–46. 10.1016/j.neuron.2015.09.020 26447571 PMC4605545

[B10] Eysenck M. W. Derakshan N. Santos R. Calvo M. G. (2007). Anxiety and cognitive performance: Attentional control theory. *Emotion* 7 336–353. 10.1037/1528-3542.7.2.336 17516812

[B11] Gustavson D. E. Miyake A. (2015). Trait worry is associated with difficulties in working memory updating. *Cogn. Emot.* 30 1289–1303. 10.1080/02699931.2015.1060194 26208534 PMC6118341

[B12] Haley A. P. Gunstad J. Cohen R. A. Jerskey B. A. Mulligan R. C. Sweet L. H. (2008). Neural correlates of visuospatial working memory in healthy young adults at risk for hypertension. *Brain Imag. Behav.* 2 192–199. 10.1007/s11682-008-9025-4

[B13] Held J. Vîslă A. Zinbarg R. E. Wolfer C. Flückiger C. (2020). How do worry and clinical status impact working memory performance? An experimental investigation. *BMC Psychiatry* 20:317. 10.1186/s12888-020-02694-x 32560680 PMC7304094

[B14] Joormann J. Gotlib I. H. (2008). Updating the Contents of Working Memory in Depression: Interference From Irrelevant Negative Material. *J. Abnorm. Psychol.* 117 182–192. 10.1037/0021-843x.117.1.182 18266496

[B15] Keller F. Hautzinger M. Kühner C. (2008). Zur faktoriellen struktur des Deutschsprachigen BDI-II. *Z. Klein. Psychol. Psychother.* 37 245–254. 10.1026/1616-3443.37.4.245

[B16] Köllner V. (2014). Psychosomatische Rehabilitation. *Psychotherapeut* 59 485–502. 10.1007/s00278-014-1085-x

[B17] Krohne H. W. Egloff B. Kohlmann C.-W. Tausch A. (1996). Untersuchungen mit einer deutschen Version der “Positive and Negative Affect Schedule” (PANAS). *Diagnostica* 42 139–156.

[B18] Li G. Huang S. Xu W. Jiao W. Jiang Y. Gao Z. (2020). The impact of mental fatigue on brain activity: a comparative study both in resting state and task state using EEG. *BMC Neurosci.* 21:20. 10.1186/s12868-020-00569-1 32398004 PMC7216620

[B19] Linden M. (2014). Psychosomatic Inpatient Rehabilitation: The German Model. *Psychother. Psychosomat.* 83 205–212. 10.1159/000358852 24970244

[B20] Mattay V. S. Fera F. Tessitore A. Hariri A. R. Berman K. F. Das S. (2006). Neurophysiological correlates of age-related changes in working memory capacity. *Neurosci. Lett.* 392 32–37. 10.1016/j.neulet.2005.09.025 16213083

[B21] McEvoy P. M. Watson H. Watkins E. R. Nathan P. (2013). The relationship between worry, rumination, and comorbidity: Evidence for repetitive negative thinking as a transdiagnostic construct. *J. Affect. Disord.* 151 313–320. 10.1016/j.jad.2013.06.014 23866301

[B22] Moran T. P. (2016). Anxiety and Working Memory Capacity: A Meta-Analysis and Narrative Review. *Psychol. Bull.* 142 831–864. 10.1037/bul0000051 26963369

[B23] Oberauer K. Süß H. M. Schulze R. Wilhelm O. Wittmann W. W. (2000). Working memory capacity - Facets of a cognitive ability construct. *Personal. Individ. Diff.* 29 1017–1045. 10.1016/s0191-8869(99)00251-2

[B24] Pfendler C. (1991). *Vergleichende Bewertung der NASA-TLX Skala und der ZEIS-Skala bei der Erfassung von Lernprozessen (Bericht 92).* Wachtberg: Forschungsinstitut für Antropotechnik.

[B25] Pinheiro J. Bates D. DebRoy S. Sarkar D. R Core Team (2021). *nlme: Linear and Nonlinear Mixed Effects Models*. *R Package Version 3.1-152.* Available online at: https://CRAN.R-project.org/package=nlme

[B26] R Core Team (2021). *R: A language and Environment for Statistical Computing.* Vienna: R Foundation for Statistical Computing.

[B27] Sari B. A. Koster E. H. W. Derakshan N. (2016). The effects of active worrying on working memory capacity. *Cogn. Emot.* 31 995–1003. 10.1080/02699931.2016.1170668 27064657

[B28] Scharfen J. Jansen K. Holling H. (2018). Retest effects in working memory capacity tests: A meta-analysis. *Psychonomic Bull. Rev.* 25 2175–2199. 10.3758/s13423-018-1461-6 29907925

[B29] Schmitz N. Arkink E. B. Mulder M. Rubia K. Admiraal-Behloul F. Schoonmann G. G. (2008). Frontal lobe structure and executive function in migraine patients. *Neurosci. Lett.* 440 92–96. 18556120 10.1016/j.neulet.2008.05.033

[B30] Snyder H. R. (2013). Major depressive disorder is associated with broad impairments on neuropsychological measures of executive function: A meta-analysis and review. *Psychol. Bull.* 139 81–132. 10.1037/a0028727 22642228 PMC3436964

[B31] Trick L. Watkins E. Windeatt S. Dickens C. (2016). The association of perseverative negative thinking with depression, anxiety and emotional distress in people with long term conditions: A systematic review. *J. Psychosom. Res.* 91 89–101. 10.1016/j.jpsychores.2016.11.004 27894469

[B32] Wagner S. Kaschel R. Paulsen S. Bleichner F. Knickenberg R. J. Beutel M. E. (2008). Does a cognitive-training programme improve the performance of middle-aged employees undergoing in-patient psychosomatic treatment? *Disab. Rehab.* 30 1786–1793. 10.1080/09638280701661380 18608414

[B33] Wagner S. Kaschel R. Paulsen S. Knickenberg R. J. Bleichner F. Beutel M. E. (2006). Kognitive Auffälligkeiten, Depressivität und Leistungsfähigkeit bei älteren Arbeitnehmern in stationärer psychosomatischer Behandlung. *Nervenarzt* 77 1338–1344. 10.1007/s00115-005-1973-y 16082527

[B34] Watkins E. R. Brown R. G. (2002). Rumination and executive function in depression: An experimental study. *J. Neurol. Neurosurg. Psychiat.* 72 400–402. 10.1136/jnnp.72.3.400 11861707 PMC1737771

[B35] World Health Organization (2004). *ICD-10: International statistical classification of diseases and related health problems: tenth revision*, 2nd Edn. Geneva: World Health Organization.

[B36] Zetsche U. Bürkner P. C. Schulze L. (2018). Shedding light on the association between repetitive negative thinking and deficits in cognitive control – A meta-analysis. *Clin. Psychol. Rev.* 63 56–65. 10.1016/j.cpr.2018.06.001 29913351

