# Supplementary material for: Examining Working Memory Performance in Adult Psychosomatic Inpatients
Source: Front Psychol. 2021 Aug 16;12:589809. doi: 10.3389/fpsyg.2021.589809 (PMC8415410; doi:10.3389/fpsyg.2021.589809)
Supplement: Supplementary file 1 [file Table_1.DOC]

**Supplementary Material**

**Additional information of the particular rehabilitation clinic**

Total sample included seventy-six inpatients (50 females) of the psychosomatic Rehabilitation Clinic Seehof in Germany which is a specialist clinic for cardiological, psychosomatic and psycho-cardiological rehabilitation. Patients’ referrals were made by the German Pension Insurance Association [“Deutsche Rentenversicherung”]. Psychosomatic rehabilitation includes a multilevel psychosomatic assessment and a multidimensional treatment focus that includes psychotherapy and different kinds of psychosocial and vocational rehabilitation (Köllner, 2016; Linden, 2014).

**Participant characteristics**

In total, 60.5% indicated being incapable of work at intake. Furthermore, during the year before the clinic intake, 46% of patients were incapable of work for six months or longer, 32% of patients were incapable of work for up to three months.

Regarding the educational level, the majority of patients (56.5%) had 10 to 12 years of school education (including vocational training), 31.5% finished high school and 9.2% had a university degree. Two participants (2.6%) only completed the mandatory school.

**Working memory task structure**

The two blocks were two alternate versions of the same updating task. Thus, the general structure of the blocks was identical (for each block 42 recall trials, 108 updating trials, taken together 150 trials) but the presented numbers and arithmetic operations were different for each block in order to avoid a possible learning effect. The WM task was self-paced and in the present study, patients needed on average 20 minutes per block.

**Data analysis.**

Reaction time values above three standard deviations of the individual mean were excluded (2% of each block) and only reaction times of correct responses were included in the analysis (Miller, 1991)[[1]](#footnote-2). All analysis were performed using R version 3.5.1 (R Core Team, 2018) and the *nlme* package (Pinheiro, Bates, DebRoy, Sarkar, & Team, 2018) for multilevel modelling.

First, to explore accuracy and reaction time in Block 1 as well as the change from Block 1 to Block 2, a multivariate growth model with a random intercept and a fixed slope was performed where the two WM blocks (WM Block 1 and Block 2) were nested at level-1 in individuals at level-2. WM block was coded with Block 1 as 0 and Block 2 as 1, with block 1 representing the baseline (Model 1). An indicator variable *h* was created indicating the type of response being analysed where *h* = 1 for accuracy (a_j_) and *h* = 2 for reaction time (rt_j_). For each outcome, the following univariate mixed model was defined:

| The value Y_hij_ for the *accuracy* outcome is: | |
| --- | --- |
| (1) | (Y_1ij_) = β_10_ + β_11_ (Block_ij_) + μ_1j_ + r_ij_ |
| The value Y_hij_ for the *reaction time* outcome is: | |
| (2) | (Y_2ij_) = β_20_ + β_21_ (Block_ij_) + μ_2j_ + r_ij_ |

Focusing on Equation (1), the indicator *h* for accuracy (*h*=1) at time i for person j is a function of the overall intercept (β_10_) which is equal to the expected mean value of accuracy when all predictors are zero (in this case when Block = 0 (Block 1)), the slope of accuracy (β_11_), representing the average rate of change in accuracy from Block 1 to Block 2 and a random effects: μ_1j_ representing person-specific differences at block1 (unique baseline values for each participant) and r_ij_, the residual error. Interpretation of the reaction time outcome in Equation (2) is identical to accuracy outcome, with the corresponding reaction time coefficients.

Next, all predictors were individually added as level-2 characteristic into Model 1. Predictors in line with research question (RQ) 2 include the total score of the Peserverative Thinking Questionnaire (PTQ) and the Becks Depression Inventory (BDI). For RQ3, age and duration of incapability of work during the last year before the clinic stay were added as predictors. All predictors were grand-mean centered and therefore, 0 represented the average value of the predictor. The following equation represents the model with the grand-mean centered PTQ as a predictor but the equation and the interpretation of all predictors is identical.

| The value Y_hij_ for the *accuracy* outcome is: | |
| --- | --- |
| (3) | (Y_1ij_) = β_10_ + β_11_ (Block_ij_) + β_12_ (PTQ) + β_15_ (Block_ij_ * PTQ) + μ_1j_ + r_ij_ |
| The value Y_hij_ for the *reaction time* outcome is: | |
| (4) | (Y_2ij_) = β_20_ + β_21_ (Block_ij_) + β_22_ (PTQ) + β_25_ (Block_ij_ * PTQ) + μ_2j_ + r_ij_ |

Focusing on Equation (1), the indicator *h* for accuracy (*h*=1) at time i for person j is a function of the overall intercept (β_10_) which is equal to the expected mean value of accuracy when all predictors are zero (in this case when Block = 0 (Block 1) and PTQ = 0 (mean PTQ score)), the slope of accuracy (β_11_), representing the average rate of change in accuracy from Block 1 to Block 2 for a patient with average PTQ score, a coefficient for PTQ (β_12_) indexing the main effect of PTQ at baseline (Block = 0), the interaction term (β_15_) for each Block by PTQ interaction, and a random effects: μ_1j_ representing person-specific differences at block1 (unique baseline values for each participant) and r_ij_, the residual error. Interpretation of the reaction time outcome in Equation (4) is identical to accuracy outcome, with the corresponding reaction time coefficients.

**References.**

Miller, J. (1991). Reaction time analysis with outlier exclusion- Bias varies with sample size.

The Quarterly Journal Of Experimental Psychology, 43(3), 907–912.

Pinheiro, J., Bates, D., DebRoy, S., Sarkar, D., & Team, R. C. (2018). nlme: Linear and Nonlinear Mixed Effects Models. Retrieved from https://cran.r-project.org/package=nlme

R Core Team. (2018). R: A language and environment for statistical computing. Vienna, Austria: R Foundation for Statistical Computing.

**Table 1A**

*Descriptive statistics and correlations for key variables*

|  |  |  | 0-order correlations | | | | | | |  |
| --- | --- | --- | --- | --- | --- | --- | --- | --- | --- | --- |
|  | **M** | ***SD*** | **1.** | **2.** | **3.** | **4.** | **5.** | **6.** | **7.** | |
| **Predictors** |  |  |  |  |  |  |  |  |  | |
| 1. Age | 52.7 | (8.4) | - |  |  |  |  |  |  | |
| 1. Duration of work incapability | 3.1 | (1.05) | - .04 | - |  |  |  |  |  | |
| 1. BDI pre | 25.9 | (12.1) | .14 | .23 | - |  |  |  |  | |
| 1. PTQ | 38.6 | (11.3) | .08 | .12 | .67*** | - |  |  |  | |
| **Dependent variables** |  |  |  |  |  |  |  |  |  | |
| 1. Accuracy Block 1 | 78.3 | (16.7) | -.25* | -.10 | -.35** | -.25* | - |  |  | |
| 1. Accuracy Block 2 | 76.1 | (11.8) | -.11 | -.11 | -.37** | -.26* | .83*** | - |  | |
| 1. Reaction time Block 1 | 3541 | (1188) | .20 | .06 | .13 | .17 | -.27* | -.23 | - | |
| 1. Reaction time Block 2 | 3410 | (1006) | .32** | .07 | .17 | .18 | -.29* | -.29* | .94*** | |

*Note.* Duration of work incapability during the last 12 months before the clinic stay (in months); PTQ = Perseverative Thinking Questionnaire; BDI = Beck Depression Inventory; pre = at intake; WM = Working memory.

* *p* < .05. ** *p* < .01. *** *p* < .001.

**Table 2A.**

*Descriptive statistics of the self-report items assessed at three time points during the experimental procedure*

|  |  | T1 | |  | T2 | | *p_(T1vsT2)_* |  | T3 | | *p_(T1vsT3)_* |
| --- | --- | --- | --- | --- | --- | --- | --- | --- | --- | --- | --- |
|  |  | *M* | *SD* |  | *M* | *SD* |  |  | *M* | *SD* |  |
|  |  |  |  |  |  |  |  |  |  |  |  |
| **PANAS items** |  |  |  |  |  |  |  |  |  |  |  |
| alert |  | 3.74 | (0.94) |  | 4.05 | (0.88) | ** |  | 4.11 | (0.93) | ** |
| interested |  | 4.08 | (0.76) |  | 4.28 | (0.84) | * |  | 4.2 | (0.99) | .28 |
| attentive |  | 3.88 | (0.78) |  | 4.09 | (0.82) | .060 |  | 3.89 | (1.05) | .91 |
| nervous |  | 2.14 | (1.1) |  | 2.36 | (1.26) | .14 |  | 1.95 | (1.15) | .22 |
| jittery |  | 1.67 | (0.91) |  | 1.97 | (1.22) | * |  | 2.03 | (1.24) | * |
| afraid |  | 1.76 | (0.98) |  | 1.51 | (0.82) | ** |  | 1.49 | (0.79) | ** |
| distressed |  | 2.04 | (1.17) |  | 1.71 | (1.03) | ** |  | 1.64 | (0.98) | ** |
|  |  |  |  |  |  |  |  |  |  |  |  |
| **Level of worry** |  | 56.17 | (24.33) |  | 43.7 | (27.83) | *** |  | 41.98 | (27.22) | *** |
|  |  |  |  |  |  |  |  |  |  |  |  |
| **NASA TLX** |  |  |  |  |  |  |  |  |  |  |  |
| mental demands |  | - | - |  | 3.75 | (0.97) | - |  | 3.84 | (0.94) | .22 |
| physical demands |  | - | - |  | 2.12 | (1.13) | - |  | 2.33 | (1.25) | * |
| temporal demands |  | - | - |  | 3.5 | (0.77) | - |  | 3.41 | (0.82) | .22 |
| effort |  | - | - |  | 3.66 | (1) | - |  | 3.87 | (0.91) | * |
| frustration |  | - | - |  | 2.17 | (1.19) | - |  | 2.11 | (1.33) | .59 |
|  |  |  |  |  |  |  |  |  |  |  |  |

*Note.* M = mean; SD = standard deviation; Higher values indicate higher scoring on the item, e.g. higher values for “alert” corresponds to being more alert; T1 = time 1 (before the start of working memory Block 1); T2 = time 2 (between working memory Block 1 and 2); T3 = time 3 (after working memory Block 2); PANAS = Positive and Negative Affect Schedule; NASA TLX = National Aeronautics and Space Administration Task Load Index.

* *p* < .05. ** *p* < .01. *** *p* < .001

1. Assumption checking showed one participant with very low accuracy and in order to check for potential influences of outlier, all analyses were rerun without this participant (*n* = 75). These analyses revealed the comparable pattern of results for accuracy as with the complete data set. [↑](#footnote-ref-2)
